# Association of air pollution with diabetic microvascular complications: A prospective cohort study

**DOI:** 10.1371/journal.pone.0354711

**Published:** 2026-08-19

**Authors:** Yuxin Nong, Huazhen Huang, Xianzhi Xu, Xin Tan, Shuai Xu, Chaoqing Wang, Yufeng Jiang, Huimin Fan, Yafeng Zhou

**Affiliations:** 1 Department of Cardiology, The Fourth Affiliated Hospital of Soochow University (Suzhou Dushu Lake Hospital), Medical Center of Soochow University, Suzhou, China; 2 Institute for Hypertension, Soochow University, Suzhou, China; 3 Institute of Advanced Computing and Digital Engineering, Shenzhen Institute of Advanced Technology, Chinese Academy of Sciences, Shenzhen, China; 4 Department of Stomatology, The Fourth Affiliated Hospital of Soochow University (Suzhou Dushu Lake Hospital), Medical Center of Soochow University, Suzhou, China; 5 Center of Translational Medicine and Clinical Laboratory, The Fourth Affiliated Hospital of Soochow University (Suzhou Dushu Lake Hospital), Medical Center of Soochow University, Suzhou, China; Nepalese Army Institute of Health Sciences, NEPAL

## Abstract

**Objective:**

To investigate the association between long-term exposure to air pollution and the risk of microvascular complications in individuals with diabetes, and provide the evidence to inform environmental strategies for prevention.

**Methods:**

The data were collected from UK Biobank cohort (n = 9,671). Kaplan-Meier curves and log-rank tests were used to compare cumulative incidence across exposure groups. Cox proportional hazards models were employed to evaluate the associations between air pollutant exposure and the development of microvascular complications, with adjusting for demographic, clinical, and biochemical covariates. Stratified and sensitivity analyses were conducted to assess the robustness of the findings. Additionally, restricted cubic splines were used to explore potential nonlinear relationships between pollutant concentrations and complication risk.

**Results:**

With a median follow-up time of 12.4 years, 2,104 participants (21.8%) developed microvascular complications. The Kaplan-Meier analysis demonstrated early divergence in cumulative incidence curves, suggesting an early impact of air pollution exposure on vascular outcomes. After multivariable adjustment, higher levels of air pollution exposure were significantly associated with increased risk of microvascular complications in patients with diabetes. Compared to the lowest quartile, participants in the highest quartile of exposure had elevated risks (NO_2_: HR = 1.27, 95% CI: 1.12–1.45; PM_10_: HR = 1.44, 95% CI: 1.27–1.64). These associations remained consistent across stratified and sensitivity analyses.

**Conclusion:**

Long-term exposure to air pollutants, particularly NO_2_ and PM_10_, is associated with an increased risk of microvascular complications among individuals with diabetes. The observed risk appears to be persistent and may begin at relatively low exposure levels, underscoring the need for preventive strategies targeting environmental risk factors.

## 1. Introduction

Diabetes has emerged as one of the most pressing global public health challenges of the 21st century. Currently, more than 580 million individuals worldwide are living with diabetes, and approximately 20% of the type 2 diabetes burden can be attributed to air pollution exposure [1]. This striking figure underscores the critical role of environmental factors in the diabetes epidemic. Over the past two decades, the health threat posed by air pollution has intensified alongside rapid global industrialization, particularly in developing countries where accelerated urbanization and industrial growth have been accompanied by severe pollution problems [2].

The air pollutants, particularly fine particulate matter (PM_X_) and nitrogen oxides (NO_X_), have been widely documented. These pollutants have been associated with diabetes in previous studies, with proposed mechanisms including insulin resistance and impaired β-cell function [3]. In addition to directly increasing the risk of diabetes, air pollutants may also be associated with the progression of diabetic microvascular complications, a group of chronic disorders that includes diabetic retinopathy, nephropathy, and peripheral neuropathy. These complications are not only highly prevalent among people with diabetes but also represent leading causes of disability and mortality in this population [4].

Epidemiological data suggest that in patients with type 2 diabetes, the prevalence of nephropathy and retinopathy can reach 25%, while peripheral neuropathy may affect over 50% of individuals [5]. These complications significantly increase the risk of serious clinical outcomes such as coronary heart disease, stroke, renal failure, and blindness [6], thereby compromising quality of life and imposing a substantial burden on healthcare systems.

From a pathophysiological perspective, air pollution may aggravate microvascular damage in diabetes through multiple mechanisms. Fine particulate matter in ambient air can accumulate in the vascular endothelium, accelerating vascular aging and injury [7]. Chronic exposure has also been linked to endothelial dysfunction and systemic inflammation, which are key processes in atherosclerosis and acute coronary events [8].

Although the potential link between air pollution and diabetic microvascular complications has begun to attract attention, current evidence remains limited. Most existing studies have focused on short-term exposures and lack comprehensive evaluations of long-term cumulative effects. More importantly, prospective data from large-scale population cohorts are scarce. To address these gaps, we utilized study data from the UK Biobank, a large-scale prospective cohort of over 500,000 participants, to systematically investigate the association between long-term air pollution exposure and the risk of diabetic microvascular complications. Our findings aim to provide a scientific basis for individualized environmental interventions in diabetes care and offer critical parameters for estimating the disease burden attributable to air pollution.

## 2. Methods

### 2.1. Study design and population

This study is based on data from the UK Biobank, a large prospective longitudinal cohort that has collected biological and medical information from over 500,000 participants. The dataset includes demographic characteristics, laboratory biomarkers, physical measurements, lifestyle factors, self-reported questionnaires, and healthcare records. The UK Biobank was established to support the prevention, diagnosis, and treatment of a wide range of life-threatening and disabling diseases. All participants provided written informed consent, and the study was approved by the NHS North West Multi-centre Research Ethics Committee (Approval number: 16/NW/0274). The present analysis was conducted using data from participants recruited between 2006 and 2010 (Application ID: 68136).

### 2.2. Air pollution exposure

Air pollution data were derived from the European-wide regulatory air quality mapping network, with a spatial resolution of 100 × 100 meters. Individual long-term exposure levels were estimated by mapping residential coordinates to these grids and applying land use regression models incorporating satellite-derived data [9]. Participants were categorized into four exposure groups (Q1–Q4) based on pollutant concentration quartiles. This exposure assessment approach—using annual average concentrations at residential addresses combined with land use regression modeling—is well-established and has been widely validated in previous UK Biobank studies [10]. To capture long-term exposure, we used the annual average concentration of NO_2_ during 2005–2007 and PM_10_ in 2007 as primary exposure indicators. We selected NO_2_ and PM_10_ as primary exposure indicators because they are the most widely measured and consistently reported pollutants in relation to cardiovascular and metabolic outcomes in the UK Biobank, and their exposure data have been extensively validated in previous studies using the same assessment framework. Other pollutants such as PM_2.5_, SO_2_, and O_3_ were not included, as they were not available or had insufficient spatial resolution in the exposure dataset for the study period. While individual time-activity patterns and residential mobility were not directly captured, such non-differential misclassification generally biases effect estimates toward the null [11], suggesting that our findings likely represent conservative estimates of the true associations.

### 2.3. Study outcome

**Fig 1:**
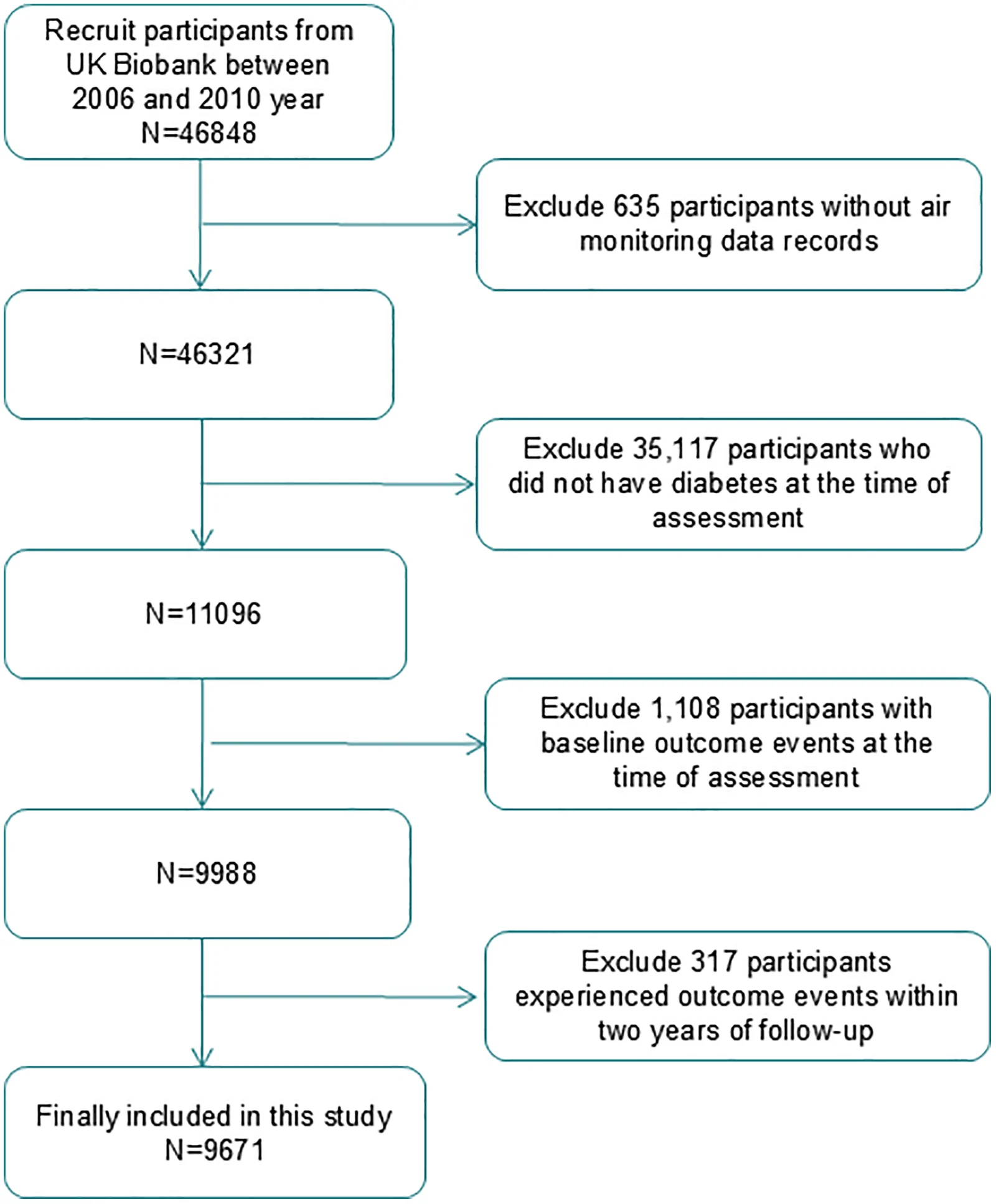
Flowchart of participant selection from the UK Biobank cohort.

The primary outcome of interest was the incidence of diabetic microvascular complications. Diabetes diagnoses and dates were obtained through UK Biobank’s linkage with hospital inpatient records, using character string mapping and subsequent conversion to ICD-10 codes (ICD-10: E10–E15). Microvascular complications were defined as the first recorded diagnosis of any diabetes-related renal, neurological, ophthalmic, or peripheral circulatory condition. Detailed diagnostic codes are provided in Supplementary Table 1 in S1 Appendix. We identified 46,848 participants with diabetes. Those with incomplete or missing data, or who developed microvascular complications within two years of recruitment, were excluded. Follow-up time was calculated from the date of recruitment to the first occurrence of a microvascular complication, death, or the censoring date. The flow chart of participant selection is shown in Fig 1.

### 2.4. Covariate assessment

Baseline covariates were collected from assessment center records and questionnaires at enrollment, including demographic variables (age, sex, ethnicity, smoking, alcohol consumption, annual income), medical history (hypertension, chronic renal failure, diabetes duration), physical measurements, and laboratory tests. The laboratory variables included: body mass index (BMI), alanine aminotransferase (ALT), aspartate aminotransferase (AST), albumin, alkaline phosphatase (ALP), C-reactive protein (CRP), serum calcium, cholesterol, creatinine, γ-glutamyl transferase (γ-GGT), glycated hemoglobin (HbA1c), phosphate, triglycerides (TG), blood urate, and vitamin D levels. Hospital diagnoses were also extracted for baseline disease assessment.

### 2.5. Statistical analysis

Continuous variables were summarized as medians with interquartile ranges (IQR), while categorical variables were presented as counts and percentages. Group differences were assessed using the Kruskal-Wallis test for continuous variables and the chi-square test for categorical variables. Kaplan–Meier curves were plotted to estimate cumulative incidence of microvascular complications across exposure groups, and differences were tested using the log-rank test. Cox proportional hazards models were used to assess the association between air pollution exposure and risk of microvascular complications, with proportional hazards assumptions checked using Schoenfeld residuals. Covariates were selected based on a priori knowledge from the literature and clinical plausibility, rather than data-driven stepwise selection. We constructed 3 Cox models. Model 1 estimated the total effect. Model 2 included established confounders. Model 3 further added biochemical markers to assess independence from metabolic status. Some of these markers may act as mediators. Model 1: adjusted only for air pollution quartile; Model 2: additionally adjusted for age, sex, BMI, ethnicity, smoking, alcohol use, annual income, HbA1c, hypertension, chronic kidney disease, and diabetes duration; Model 3: further adjusted for biochemical and inflammatory markers including AST, ALT, ALP, CRP, serum calcium, cholesterol, creatinine, γ-GGT, phosphate, triglycerides, blood urate, and vitamin D. To examine effect modification, stratified analyses were performed by age, sex, BMI, and diabetes duration.

Potential nonlinear exposure-response relationships were explored using restricted cubic spline functions. Sensitivity analyses were conducted by excluding participants with less than three years of follow-up to minimize reverse causality and potential bias.

## 3. Results

### 3.1. Baseline characteristics of the study population

Among the included participants, 5,858 (60.6%) were men and 3,813 (39.4%) were women. During the follow-up period, 2,104 individuals (21.8%) developed microvascular complications. Baseline characteristics by complication status are shown in Table 1. The median age in both groups was 62 years (IQR: 56–66 in the non-complication group; 57–66 in the complication group). Participants who developed complications had significantly higher BMI (31.36 vs. 30.54 kg/m^2^, P < 0.001) and HbA1c levels (56.45% vs. 50.20%, P < 0.001). The prevalence of hypertension (75.0% vs. 70.4%, P = 0.021) and chronic renal failure (7.2% vs. 5.4%, P < 0.001) was also higher in the complication group. Baseline characteristics by quartile-based exposure groups of NO_2_ and PM_10_ are presented in Supplementary Table 2 and Supplementary Table 3 in S1 Appendix. A higher proportion of women was observed in the highest PM_10_ exposure group (P = 0.01), while no significant sex difference was found across NO_2_ quartiles (P = 0.412). Notably, the proportion of White participants decreased significantly in high exposure groups, with corresponding increases in Asian and Black populations (P < 0.001). Lifestyle factors such as smoking and drinking status also varied significantly across exposure categories (P < 0.001). Clinically, NO_2_ exposure was associated with elevated CRP levels (P = 0.014), and both pollutants were significantly associated with variations in serum creatinine (P < 0.05). Strikingly, vitamin D levels were significantly lower in the highest PM_10_ quartile (P < 0.001 for both NO_2_ and PM_10_), suggesting meaningful associations between air pollution exposure and multiple demographic and clinical indicators.

**Table 1:** Baseline characteristics classified according to the microvascular complications of the participants.

Variables	Microvascular complications	
	No	Yes
Age (year)	62.00 [56.00, 66.00]	62.00 [57.00, 66.00]
Sex (%)		
Female	2992 (39.5)	821 (39.0)
Male	4575 (60.5)	1283 (61.0)
Ethnic (%)		
White	6584 (87.0)	1782 (84.7)
Mixed	50 (0.7)	9 (0.4)
Asian or Asian British	518 (6.8)	157 (7.5)
Black or Black British	220 (2.9)	70 (3.3)
Other	195 (2.6)	86 (4.1)
Alcohol (%)		
Never	715 (9.4)	207 (9.8)
Previous	670 (8.9)	192 (9.1)
Current	6117 (80.8)	1679 (79.8)
Unknown	65 (0.9)	26 (1.2)
Smoking (%)		
No	6607 (87.3)	1840 (87.5)
Most or all days	693 (9.2)	181 (8.6)
Occasionally	218 (2.9)	64 (3.0)
Unknown	49 (0.6)	19 (0.9)
BMI (Kg/m^2^)	30.54 [27.19, 34.70]	31.36 [27.77, 35.54]
ALT (U/L)	23.90 [17.91, 33.04]	23.88 [17.80, 33.08]
AST (U/L)	24.90 [20.70, 30.70]	24.60 [20.30, 31.10]
Albumin (g/L)	44.79 [42.90, 46.62]	44.32 [42.42, 46.34]
ALP (U/L)	86.00 [71.30, 104.60]	88.35 [73.10, 106.75]
CRP (median [IQR])	1.90 [0.92, 4.08]	2.22 [1.02, 4.62]
Serum calcium (mmol/L)	2.38 [2.32, 2.45]	2.38 [2.32, 2.45]
Cholesterol (mmol/L)	4.34 [3.73, 5.03]	4.21 [3.67, 4.92]
Creatinine (umol/L)	72.40 [61.60, 84.95]	74.05 [61.80, 88.30]
γGGT (U/L)	34.80 [23.60, 55.95]	36.70 [23.90, 59.92]
HbA1c (%)	50.20 [42.90, 59.50]	56.45 [48.40, 66.62]
Phosphate (mmol/L)	1.15 [1.03, 1.26]	1.14 [1.03, 1.25]
Triglycerides (mmol/L)	1.79 [1.23, 2.58]	1.89 [1.27, 2.70]
Blood urate (umol/L)	5.59 [4.65, 6.71]	5.82 [4.81, 7.23]
Vitamin D (nmol/L)	39.60 [26.90, 56.80]	38.40 [26.20, 53.90]
Hypertension (%)		
No	2239 (29.6)	527 (25.0)
Yes	5328 (70.4)	1577 (75.0)
Chronic renal failure (%)		
No	7160 (94.6)	1953 (92.8)
Yes	407 (5.4)	151 (7.2)
Course of diabetes (%)		
< 3	3751 (49.6)	768 (36.5)
3 ~ 5	1463 (19.3)	396 (18.8)
5 ~ 10	1993 (26.3)	742 (35.3)
≥ 10	360 (4.8)	198 (9.4)
Annual income (%)		
£18,000 ~ £30999	2623 (34.7)	814 (38.7)
£31000 ~ £51999	1736 (22.9)	483 (23.0)
£52000 ~ £100000	1136 (15.0)	279 (13.3)
≥ £100000	573 (7.6)	122 (5.8)
Unknown	1499 (19.8)	406 (19.3)

BMI: Body mass index; ALT: Alanine aminotransferase; AST: Aspartate aminotransferase; ALP: Alkaline phosphatase; γGGT: Gamma-glutamyl transferase; HbA1c: Glycated hemoglobin; CRP: C-reactive protein.

### 3.2. Cumulative risk of microvascular complications by air pollution exposure

**Fig 2:**
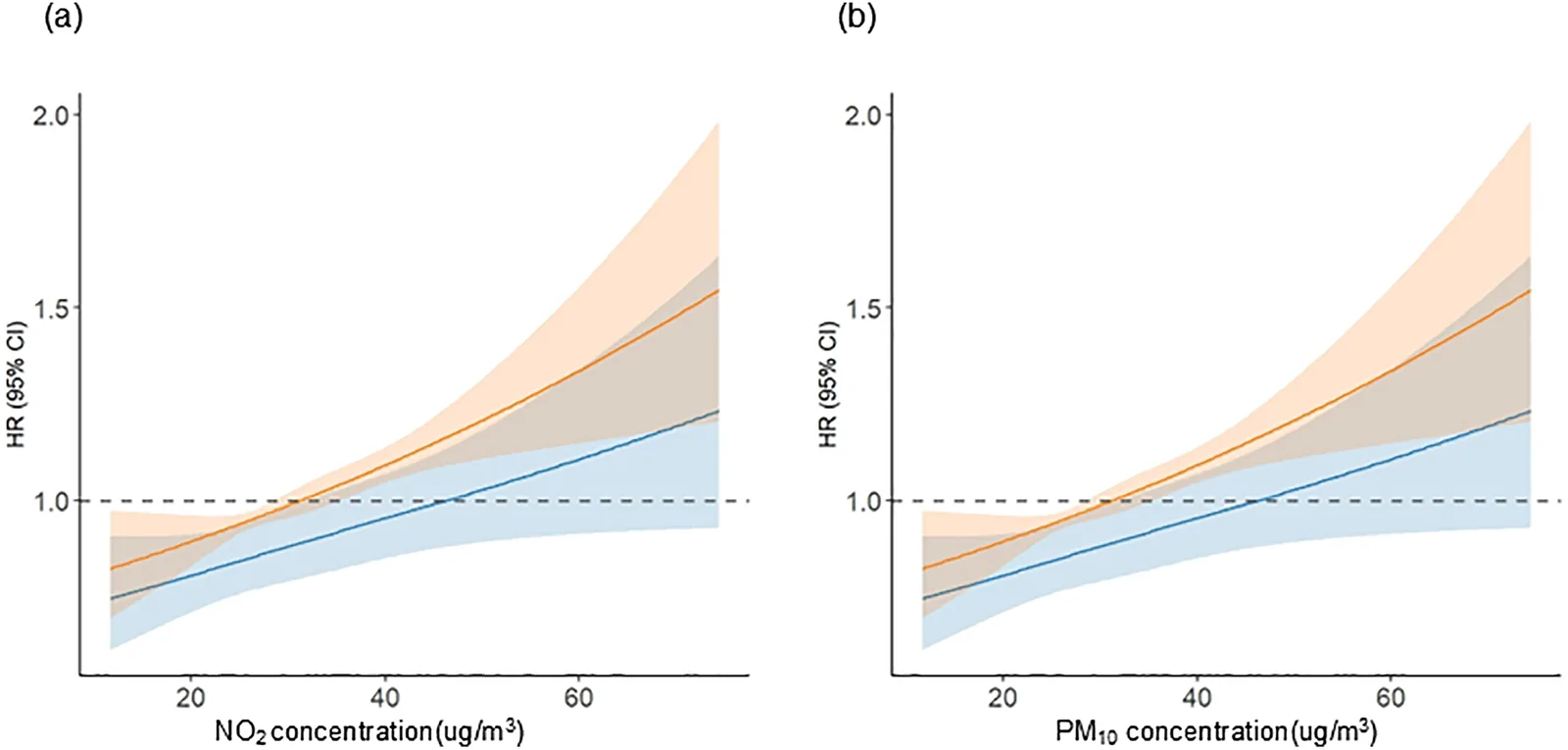
Cumulative Risk curves of diabetic microvascular complications in different air pollution exposure groups. (a) NO_2_ (b) PM_10_.

Kaplan–Meier analyses (Fig 2a–b) demonstrated significant relationships between exposure to both NO_2_ and PM_10_ and the cumulative incidence of microvascular complications. For NO_2_, the highest exposure group (Q4) showed a consistently elevated cumulative risk compared to the lowest group (Q1) throughout the follow-up period (log-rank P < 0.001), with early divergence of risk curves suggesting early emergence of exposure-related risk differentials. A similar but more pronounced pattern was observed for PM_10_ exposure. The Q4 group had the steepest incidence curve (log-rank P < 0.001), with earlier and wider separation from lower exposure groups, suggesting a potentially stronger risk elevation. For both pollutants, significant divergence appeared within two years of follow-up, with risk differences widening over time.

### 3.3. Association between pollutant exposure and risk of microvascular complications

Cox proportional hazards models showed significant associations between both NO_2_ and PM_10_ exposure and complication risk (Table 2). In the unadjusted Model 1, the highest NO_2_ quartile (Q4) was associated with increased risk (HR = 1.31, 95% CI: 1.16–1.48, P < 0.001), with a borderline significant increase in Q3 (HR = 1.13; 95% CI: 1.00–1.28; P = 0.05), but no significant risk in Q2 (HR = 1.03, 95% CI: 0.90–1.16, P = 0.695). PM_10_ exposure showed a clearer risk gradient, with significantly elevated risk beginning in Q2 (HR = 1.20, 95% CI: 1.06–1.36; P = 0.005) and peaking in Q4 (HR = 1.46, 95% CI: 1.29–1.65, P < 0.001). These associations persisted after adjusting for confounders in Models 2 and 3. In the fully adjusted Model 3, NO_2_ Q4 remained significantly associated with increased risk (HR = 1.27; 95% CI: 1.12–1.45; P < 0.001), while PM_10_ Q4 demonstrated a stronger association (HR = 1.44; 95% CI: 1.27–1.64; P < 0.001). PM_10_ exposure showed stronger and earlier risk elevations than NO_2_, suggesting broader health impacts even at lower levels. Further subtype analyses showed that the associations of NO_2_ and PM_10_ exposure with different outcomes (nephropathy, retinopathy, and neuropathy) were generally consistent in terms of direction and trend, although the effect estimates appeared somewhat stronger for nephropathy than for the other two endpoints (Supplementary Table 4 in S1 Appendix).

**Table 2:** Cox regression analysis between air exposure and diabetic microvascular complications.

	Model 1	Model 2	Model 3
	HR (95%CI) P-value	HR (95%CI) P-value	HR (95%CI) P-value
NO2			
Q2	1.03 (0.90–1.16) 0.695	1.03 (0.91–1.17) 0.661	1.01 (0.89–1.15) 0.826
Q3	1.13 (1.00–1.28) 0.05	1.10 (0.98–1.25) 0.116	1.10 (0.97–1.24) 0.152
Q4	1.31 (1.16–1.48) <0.001	1.28 (1.13–1.46) <0.001	1.27 (1.12–1.45) <0.001
PM10			
Q2	1.20 (1.06–1.36) 0.005	1.21 (1.06–1.37) 0.004	1.21 (1.06–1.38) 0.003
Q3	1.28 (1.13–1.45) <0.001	1.29 (1.14–1.46) <0.001	1.29 (1.14–1.46) <0.001
Q4	1.46 (1.29–1.65) <0.001	1.45 (1.27–1.64) <0.001	1.44 (1.27–1.64) <0.001

Model 1: adjusted only for air pollution quartile;

Model 2: adjusted for age, sex, BMI, ethnicity, smoking, alcohol use, annual income, HbA1c, hypertension, chronic kidney disease, and diabetes duration;

Model 3: adjusted for Model 2 plus AST, ALT, ALP, CRP, serum calcium, cholesterol, creatinine, γ-GGT, phosphate, triglycerides, blood urate, and vitamin D.

### 3.4. Subgroup analyses

To further assess the differences in the impact of air pollutants on different groups of people, stratified analyses were conducted by age (<60/ ≥ 60 years), sex, BMI (<30/ ≥ 30 kg/m^2^), and diabetes duration (<5/ ≥ 5 years). Although none of the interaction terms reached statistical significance (all P-interaction > 0.05), numeric differences in risk estimates were observed (Table 3). In age-stratified models, individuals ≥60 years had slightly higher risk in the NO_2_ Q4 group (HR = 1.31; 95% CI: 1.12–1.53) compared to those <60 years (HR = 1.26; 95% CI: 1.01–1.57), though P_-interaction_ = 0.784. A similar trend was seen for PM_10_. By sex, adjusted risks for NO_2_ were higher in women (HR = 1.36; 95% CI: 1.10–1.67) than in men (HR = 1.24; 95% CI: 1.06–1.47), but interaction P = 0.332. PM_10_ showed comparable effects across sexes. For BMI, individuals with BMI ≥ 30 showed higher HRs for both pollutants, but interaction terms remained non-significant. Regarding diabetes duration, NO_2_ had a greater impact in those with <5 years since diagnosis, whereas PM_10_ had stronger effects in the ≥ 5-year group. However, interactions were marginal (P = 0.08 and 0.071, respectively).

**Table 3:** Stratified analyses of air pollution exposure and risk of microvascular complications by age, sex, BMI, and diabetes duration.

Subgroup	Model 1		Model 2		Model 3	
	HR (95%CI) P-value	P for interaction	HR (95%CI) P-value	P for interaction	HR (95%CI) P-value	P for interaction
NO_2_						
Age		0.784		0.891		0.908
<60						
Q2	0.97 (0.77–1.21) 0.776		1.01 (0.81–1.27) 0.915		1.03 (0.82–1.29) 0.813	
Q3	1.12 (0.90–1.39) 0.309		1.14 (0.91–1.42) 0.257		1.14 (0.91–1.42) 0.245	
Q4	1.25 (1.02–1.54) 0.032		1.26 (1.01–1.57) 0.037		1.26 (1.01–1.57) 0.041	
≥60						
Q2	1.06 (0.91–1.24) 0.44		1.04 (0.90–1.22) 0.577		1.02 (0.88–1.19) 0.777	
Q3	1.14 (0.99–1.33) 0.078		1.09 (0.94–1.27) 0.247		1.09 (0.93–1.27) 0.279	
Q4	1.38 (1.19–1.60) <0.001		1.32 (1.13–1.54) <0.001		1.31 (1.12–1.53) <0.001	
Sex		0.387		0.151		0.332
Male						
Q2	1.04 (0.89–1.22) 0.619		1.05 (0.89–1.23) 0.583		1.04 (0.88–1.22) 0.668	
Q3	1.18 (1.01–1.39) 0.035		1.15 (0.98–1.35) 0.083		1.14 (0.97–1.34) 0.11	
Q4	1.32 (1.13–1.54) <0.001		1.25 (1.06–1.47) 0.007		1.24 (1.06–1.47) 0.009	
Female						
Q2	1.00 (0.82–1.22) 0.998		1.02 (0.83–1.25) 0.86		1.01 (0.83–1.24) 0.908	
Q3	1.06 (0.87–1.29) 0.589		1.06 (0.87–1.29) 0.572		1.05 (0.86–1.28) 0.647	
Q4	1.30 (1.08–1.58) 0.007		1.36 (1.11–1.66) 0.004		1.36 (1.10–1.67) 0.004	
BMI		0.509		0.175		0.12
<30						
Q2	1.03 (0.84–1.25) 0.798		1.03 (0.84–1.26) 0.756		1.01 (0.83–1.23) 0.928	
Q3	1.19 (0.98–1.45) 0.08		1.18 (0.97–1.44) 0.105		1.17 (0.96–1.43) 0.12	
Q4	1.33 (1.11–1.61) 0.003		1.23 (1.00–1.51) 0.045		1.24 (1.01–1.51) 0.042	
≥30		0.07				
Q2	1.02 (0.87–1.19) 0.84		1.02 (0.87–1.20) 0.801		1.01 (0.86–1.19) 0.904	
Q3	1.08 (0.92–1.26) 0.346		1.06 (0.91–1.25) 0.446		1.05 (0.89–1.23) 0.561	
Q4	1.31 (1.12–1.53) <0.001		1.34 (1.14–1.58) <0.001		1.33 (1.13–1.56) <0.001	
Course of diabetes				0.352		0.08
<5						
Q2	1.03 (0.87–1.23) 0.696		1.05 (0.89–1.25) 0.561		1.03 (0.87–1.23) 0.704	
Q3	1.11 (0.94–1.32) 0.216		1.10 (0.93–1.31) 0.249		1.09 (0.92–1.29) 0.338	
Q4	1.42 (1.21–1.67) <0.001		1.38 (1.17–1.64) <0.001		1.35 (1.13–1.60) <0.001	
≥5						
Q2	1.02 (0.84–1.23) 0.87		1.02 (0.85–1.23) 0.813		1.01 (0.83–1.22) 0.935	
Q3	1.14 (0.95–1.36) 0.168		1.13 (0.94–1.35) 0.207		1.12 (0.93–1.34) 0.234	
Q4	1.17 (0.97–1.40) 0.093		1.17 (0.97–1.42) 0.108		1.18 (0.97–1.43) 0.099	
PM10						
Age						
<60		0.838		0.904		0.934
Q2	1.07 (0.85–1.35) 0.54		1.08 (0.86–1.36) 0.523		1.08 (0.85–1.36) 0.535	
Q3	1.35 (1.08–1.68) 0.008		1.33 (1.07–1.66) 0.012		1.34 (1.07–1.67) 0.009	
Q4	1.39 (1.13–1.73) 0.002		1.39 (1.11–1.74) 0.004		1.42 (1.14–1.78) 0.002	
≥60						
Q2	1.28 (1.10–1.49) 0.002		1.27 (1.09–1.48) 0.002		1.28 (1.09–1.49) 0.002	
Q3	1.25 (1.08–1.46) 0.004		1.27 (1.08–1.48) 0.003		1.27 (1.09–1.48) 0.003	
Q4	1.54 (1.32–1.79) <0.001		1.48 (1.26–1.73) <0.001		1.48 (1.26–1.73) <0.001	
Sex						0.576
Male						
Q2	1.26 (1.07–1.48) 0.005	0.457	1.27 (1.08–1.49) 0.004	0.265	1.28 (1.09–1.51) 0.003	
Q3	1.29 (1.09–1.51) 0.002		1.30 (1.11–1.53) 0.001		1.32 (1.12–1.55) <0.001	
Q4	1.56 (1.34–1.83) <0.001		1.51 (1.28–1.78) <0.001		1.52 (1.29–1.79) <0.001	
Female						
Q2	1.11 (0.90–1.36) 0.332		1.10 (0.90–1.36) 0.355		1.09 (0.88–1.34) 0.431	
Q3	1.26 (1.03–1.54) 0.022		1.27 (1.04–1.54) 0.021		1.26 (1.03–1.54) 0.024	
Q4	1.32 (1.08–1.61) 0.006		1.34 (1.09–1.64) 0.006		1.32 (1.08–1.63) 0.008	
BMI		0.111		0.119		0.111
<30						
Q2	1.24 (1.02–1.52) 0.034		1.18 (0.97–1.45) 0.099		1.19 (0.97–1.45) 0.097	
Q3	1.30 (1.07–1.59) 0.009		1.28 (1.05–1.57) 0.016		1.27 (1.04–1.56) 0.02	
Q4	1.44 (1.19–1.75) <0.001		1.33 (1.09–1.64) 0.006		1.34 (1.09–1.65) 0.005	
≥30						
Q2	1.17 (0.99–1.38) 0.061		1.22 (1.04–1.44) 0.018		1.22 (1.04–1.44) 0.018	
Q3	1.25 (1.07–1.47) 0.006		1.28 (1.09–1.51) 0.002		1.29 (1.10–1.52) 0.002	
Q4	1.49 (1.27–1.75) <0.001		1.55 (1.31–1.82) <0.001		1.54 (1.31–1.82) <0.001	
Course of diabetes		0.072		0.384		0.071
<5						
Q2	1.11 (0.93–1.31) 0.25		1.13 (0.95–1.34) 0.171		1.14 (0.96–1.36) 0.137	
Q3	1.25 (1.06–1.47) 0.009		1.26 (1.06–1.48) 0.007		1.25 (1.06–1.48) 0.008	
Q4	1.44 (1.22–1.69) <0.001		1.41 (1.19–1.68) <0.001		1.39 (1.17–1.65) <0.001	
≥5						
Q2	1.31 (1.08–1.58) 0.006		1.31 (1.08–1.58) 0.006		1.31 (1.08–1.59) 0.006	
Q3	1.32 (1.09–1.60) 0.004		1.34 (1.10–1.62) 0.003		1.34 (1.10–1.62) 0.003	
Q4	1.48 (1.23–1.78) <0.001		1.49 (1.23–1.81) <0.001		1.51 (1.24–1.83) <0.001	

Model 1: adjusted only for air pollution quartile;

Model 2: adjusted for age, sex, BMI, ethnicity, smoking, alcohol use, annual income, HbA1c, hypertension, chronic kidney disease, and diabetes duration;

Model 3: adjusted for Model 2 plus AST, ALT, ALP, CRP, serum calcium, cholesterol, creatinine, γ-GGT, phosphate, triglycerides, blood urate, and vitamin D.

### 3.5. Nonlinear associations between air pollution and microvascular complications

**Fig 3:**
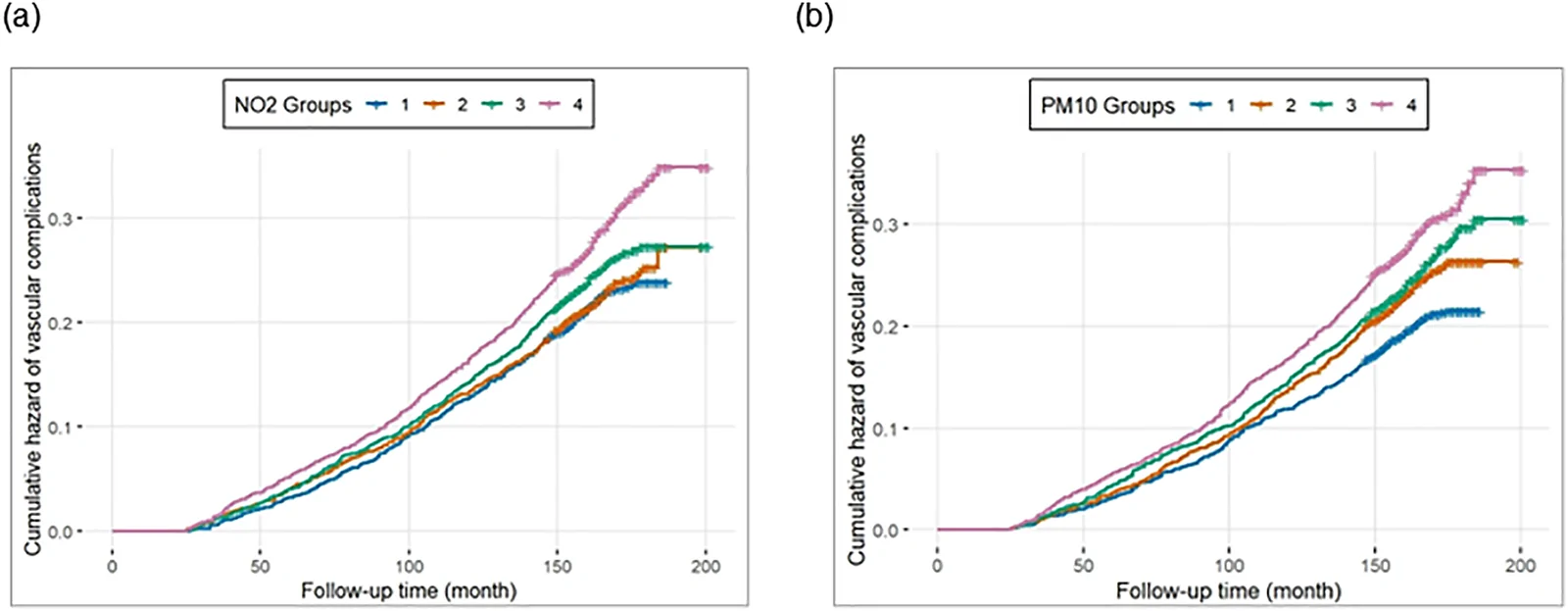
Restricted cubic splines of air pollution and diabetic microvascular complication events. (a) NO_2_ (b) PM_10_.

Restricted cubic spline models revealed nonlinear associations between pollutant exposure and complication risk, as shown in Fig 3. For NO_2_, risk remained flat below 20 μg/m^3^ but rose sharply above 25 μg/m^3^, suggesting a potential threshold effect. In contrast, PM_10_ exhibited increasing risk even at low concentrations (15–25 μg/m^3^), with sustained high risk at higher levels. These patterns were consistent across both unadjusted and fully adjusted models, although effect estimates were attenuated after adjustment, indicating partial confounding. These findings highlight a concentration-dependent relationship, especially for PM_10_, and underscore the need for tailored pollution control strategies in diabetes populations.

### 3.6. Sensitivity analyses

To assess model robustness, sensitivity analyses excluded participants with follow-up ≤3 years. Results were consistent with the primary analysis, as shown in Table 4. In unadjusted models, the highest NO_2_ and PM_10_ quartiles were associated with elevated risks (NO_2_ Q4: HR = 1.28, 95% CI: 1.13–1.45, P < 0.001, PM_10_ Q4: HR = 1.46, 95% CI: 1.28–1.65, P < 0.001). In fully adjusted models (Model 3), risk estimates remained stable (NO_2_ Q4: HR = 1.24, 95% CI: 1.09–1.42, P = 0.001, PM_10_ Q4: HR = 1.45, 95% CI: 1.27–1.65, P < 0.001). All effect size differences between main and sensitivity analyses were <5%, and directions of association remained unchanged. These consistent results suggest strong robustness to confounding adjustment and confirm the reliability of the observed associations between long-term air pollution exposure and diabetic microvascular complications.

**Table 4:** Sensitivity analyses for the association between long-term air pollution exposure and diabetic microvascular complications.

	Model 1	Model 2	Model 3
	HR (95%CI) P-value	HR (95%CI) P-value	HR (95%CI) P-value
NO2			
Q2	0.99 (0.87–1.13) 0.926	1.00 (0.88–1.14) 0.978	0.98 (0.86–1.12) 0.809
Q3	1.11 (0.97–1.25) 0.119	1.08 (0.95–1.23) 0.236	1.07 (0.94–1.22) 0.29
Q4	1.28 (1.13–1.45) <0.001	1.25 (1.10–1.43) <0.001	1.24 (1.09–1.42) 0.001
PM10			
Q2	1.21 (1.06–1.38) 0.004	1.22 (1.07–1.39) 0.003	1.23 (1.07–1.40) 0.002
Q3	1.29 (1.13–1.46) <0.001	1.30 (1.14–1.48) <0.001	1.30 (1.14–1.48) <0.001
Q4	1.46 (1.28–1.65) <0.001	1.44 (1.27–1.65) <0.001	1.45 (1.27–1.65) <0.001

Model 1: adjusted only for air pollution quartile;

Model 2: adjusted for age, sex, BMI, ethnicity, smoking, alcohol use, annual income, HbA1c, hypertension, chronic kidney disease, and diabetes duration;

Model 3: adjusted for Model 2 plus AST, ALT, ALP, CRP, serum calcium, cholesterol, creatinine, γ-GGT, phosphate, triglycerides, blood urate, and vitamin D.

## 4. Discussion

Diabetic microvascular complications—including nephropathy, retinopathy, and peripheral neuropathy—remain leading causes of disability and mortality among individuals with diabetes. In this study based on 9,671 diabetic participants from the UK Biobank, we systematically assessed the association between long-term exposure to air pollutants (NO_2_ and PM_10_) and the risk of microvascular complications over a follow-up period of up to 16.7 years. Our findings showed a significant positive association between long-term exposure to NO_2_ and PM_10_ and the risk of developing diabetic microvascular complications, even after adjustment for demographic characteristics, lifestyle factors, metabolic indicators, and inflammatory biomarkers. These results may have critical clinical and public health implications, as air pollution is a modifiable environmental factor that warrants consideration as a potential target for preventing diabetes-related complications.

Previous studies have indicated that NO_2_, a major traffic-related air pollutant, is independently associated with increased risks of all-cause, cardiovascular, and respiratory mortality, irrespective of other pollutants [12]. Mechanistically, based on previous experimental evidence, NO_2_ has been suggested to induce oxidative stress by generating free radicals, which may contribute to structural damage in cells and abnormal intracellular signaling, or by affecting mitochondrial function [13,14]. Similarly, particulate matter such as PM_10_ is a well-recognized environmental hazard. Our results suggest that PM_10_, like NO_2_, exerts considerable microvascular toxicity and elevates the risk of diabetic complications. Evidence from cohort studies has linked these pollutants with increased incidence of aortic dissection and cardiovascular mortality, potentially through mechanisms involving endothelial dysfunction and vascular smooth muscle remodeling. Notably, several recent large-scale UK Biobank-based studies have consistently reported positive associations between long-term air pollution exposure and diabetic microvascular complications, with hazard ratios ranging from 1.06 to 1.15 per interquartile range increase in PM_2.5_, PM_10_, and NO_2_ [15,16]. Our observed effect estimates (HRs up to 1.44 for PM_10_ Q4) are slightly higher than those reported in some previous studies, which may be attributed to our longer follow-up duration (up to 16.7 years). Furthermore, our study extends previous findings in several aspects: PM_10_ showed earlier (from Q2) and stronger effects than NO_2_; restricted cubic splines revealed a threshold effect for NO_2_ (~25 μg/m^3^) versus a nearly linear risk for PM_10_; the extended follow-up (up to 16.7 years) captured early divergence of risk curves within two years; and subgroup analyses consistently suggested higher susceptibility in older adults, females, and obese individuals. These distinctive features position our study as a meaningful complement to the existing evidence base for air pollution and diabetic microvascular complications.

The biological plausibility linking air pollutant exposure to diabetic microvascular complications is strongly supported by experimental and clinical evidence. Mechanistically, both NO_2_ and PM_10_ are potent inducers of systemic inflammation and oxidative stress. Once inhaled, these pollutants can trigger a cascade of inflammatory responses, reflected in our observed elevation of C-reactive protein (CRP) levels in higher exposure groups [17]. At the cellular level, these pollutants promote the overproduction of reactive oxygen species (ROS) and reactive nitrogen species (RNS), overwhelming endogenous antioxidant defenses. In diabetes, chronic hyperglycemia itself establishes a self-perpetuating vicious cycle with oxidative stress: hyperglycemia enhances ROS production through mitochondrial dysfunction, while excessive ROS further impairs insulin signaling and β-cell function, creating a feedback loop that amplifies tissue damage [18]. In particular, NO_2_ exposure can induce nitro-oxidative stress, a process that may synergize with the “metabolic memory” phenomenon in diabetes—the persistent vascular damage even after glycemic control is achieved—by perpetuating epigenetic modifications that sustain pro-inflammatory gene expression [19]. Furthermore, pollutant-induced mitochondrial dysfunction not only impairs cellular energy metabolism but also amplifies ROS generation, creating a vicious cycle of oxidative damage. Over time, these molecular insults—inflammation, oxidative/nitro-oxidative stress, and mitochondrial impairment—can drive epigenetic alterations (e.g., DNA methylation, histone modifications) in vascular endothelial cells, smooth muscle cells, and podocytes, ultimately predisposing to the development and progression of organ-specific microvascular injuries, including retinopathy, nephropathy, and neuropathy [20]. Our findings provide an epidemiological link that aligns with previously proposed mechanistic pathways.

In diabetic individuals, vascular injury is often exacerbated by glucolipotoxicity. Even in the absence of air pollution, the progression of microvascular complications is almost inevitable as the disease advances [21]. However, chronic exposure to air pollution may substantially accelerate this trajectory, further compromising patients’ quality of life and increasing healthcare burdens.

Although our stratified analyses did not detect statistically significant effect modification by age, sex, BMI, or diabetes duration, we observed trends indicating a potentially stronger adverse effect of air pollution in subgroups such as older adults (≥60 years), females, individuals with obesity (BMI ≥ 30), and those with longer disease duration (except for NO_2_). These findings may reflect increased vascular vulnerability in these populations, amplifying the detrimental effects of pollutant exposure.

Several limitations should be acknowledged. Selection bias may exist due to the UK Biobank’s volunteer-based recruitment, which tends to enroll healthier and more socioeconomically advantaged individuals, potentially limiting generalizability to broader populations. Moreover, which may limit the generalizability of our findings to other air pollutants such as PM_2.5_, SO_2_, and O_3_. However, NO_2_ and PM_10_ are recognized as major traffic-related pollutants with well-established health effects, and our findings provide a solid basis for future investigations incorporating a broader range of pollutants. Furthermore, despite rigorous adjustment for a wide range of confounders, residual confounding by unmeasured variables—including physical activity, dietary patterns, and occupational exposure—cannot be entirely excluded. In addition, the use of baseline residential addresses without accounting for residential mobility may introduce non-differential exposure misclassification, which typically biases effect estimates toward the null. Finally, some biomarkers adjusted in Model 3 (e.g., CRP, lipids) may lie on the causal pathway, and controlling for these potential mediators could represent over-adjustment, yielding conservative estimates. Nonetheless, the consistent positive associations across Models 1–3 support the robustness of our findings.

## 5. Conclusion

Our study provides robust evidence that long-term exposure to ambient air pollution—specifically NO_2_ and PM_10_—is associated with increased risk of microvascular complications in patients with diabetes. These associations were observable even among participants with shorter exposure duration and appeared to strengthen with longer follow-up time. Our findings provide information that may be useful for integrating air pollution control into diabetes management strategies and offer a scientific rationale for developing personalized environmental interventions targeting high-risk diabetic populations.

## Supporting information

S1 Appendix Supplementary tables including ICD-10 diagnostic codes for diabetic microvascular complications, participant baseline characteristics stratified by NO_2_ and PM_10_ exposure quartiles, and subtype-specific associations between air pollutants and nephropathy, retinopathy and peripheral neuropathy. (DOC)
