# Supplementary material for: Association of air pollution with diabetic microvascular complications: A prospective cohort study
Source: PLoS One. 2026 Aug 19;21(8):e0354711. doi: 10.1371/journal.pone.0354711 (PMC13489485; doi:10.1371/journal.pone.0354711)
Supplement: Supplementary file 1 [file pone.0354711.s001.doc]

Supplementary Table 1. ICD-10 diagnostic code for diabetic microvascular complications.

| Code |  |
| --- | --- |
| E10.2 | Insulin-dependent diabetes mellitus with renal complications |
| E10.3 | Insulin-dependent diabetes mellitus with ophthalmic complications |
| E10.4 | Insulin-dependent diabetes mellitus with neurological complications |
| E10.5 | Insulin-dependent diabetes mellitus with peripheral circulatory complications |
| E11.2 | Non-insulin-dependent diabetes mellitus with renal complications |
| E11.3 | Non-insulin-dependent diabetes mellitus with ophthalmic complications |
| E11.4 | Non-insulin-dependent diabetes mellitus with neurological complications |
| E11.5 | Non-insulin-dependent diabetes mellitus with peripheral circulatory complications |
| E13.2 | Other specified diabetes mellitus with renal complications |
| E13.3 | Other specified diabetes mellitus with ophthalmic complications |
| E13.4 | Other specified diabetes mellitus with neurological complications |
| E13.5 | Other specified diabetes mellitus with peripheral circulatory complications |
| E14.2 | Unspecified diabetes mellitus with renal complications |
| E14.3 | Unspecified diabetes mellitus with ophthalmic complications |
| E14.4 | Unspecified diabetes mellitus with neurological complications |
| E14.5 | Unspecified diabetes mellitus with peripheral circulatory complications |

Supplementary Table 2. Baseline characteristics classified according to the quartile of nitrogen dioxide concentration.

| Variables | Q1 | Q2 | Q3 | Q4 | P-Value |
| --- | --- | --- | --- | --- | --- |
| Age (year) | 62.00 [57.00, 66.00] | 62.00 [56.00, 66.00] | 62.00 [56.00, 66.00] | 61.00 [54.00, 65.00] | <0.001 |
| Sex (%) |  |  |  |  | 0.412 |
| Female | 923 (38.2) | 949 (39.2) | 979 (40.5) | 962 (39.8) |  |
| Male | 1494 (61.8) | 1469 (60.8) | 1439 (59.5) | 1455 (60.2) |  |
| Ethnic (%) |  |  |  |  | <0.001 |
| White | 2333 (96.5) | 2279 (94.3) | 2149 (88.9) | 1605 (66.4) |  |
| Mixed | 10 (0.4) | 14 (0.6) | 12 (0.5) | 23 (1.0) |  |
| Asian or Asian British | 37 (1.5) | 72 (3.0) | 148 (6.1) | 418 (17.3) |  |
| Black or Black British | 11 (0.5) | 18 (0.7) | 51 (2.1) | 210 (8.7) |  |
| Other | 26 (1.1) | 35 (1.4) | 58 (2.4) | 161 (6.7) |  |
| Alcohol (%) |  |  |  |  | <0.001 |
| Never | 154 (6.4) | 187 (7.7) | 212 (8.8) | 369 (15.3) |  |
| Previous | 169 (7.0) | 221 (9.1) | 255 (10.5) | 217 (9.0) |  |
| Current | 2082 (86.1) | 2000 (82.7) | 1926 (79.7) | 1787 (73.9) |  |
| unknow | 12 (0.5) | 10 (0.4) | 25 (1.0) | 44 (1.8) |  |
| Smoking (%) |  |  |  |  | <0.001 |
| No | 2175 (90.0) | 2133 (88.2) | 2093 (86.6) | 2045 (84.6) |  |
| Most or all days | 183 (7.6) | 214 (8.9) | 231 (9.6) | 246 (10.2) |  |
| Occasionally | 55 (2.3) | 65 (2.7) | 73 (3.0) | 89 (3.7) |  |
| unknow | 4 (0.2) | 6 (0.2) | 21 (0.9) | 37 (1.5) |  |
| BMI (Kg/m²) | 30.64 [27.35, 34.62] | 30.81 [27.53, 34.87] | 31.21 [27.53, 35.48] | 30.27 [26.89, 34.68] | <0.001 |
| ALT (U/L) | 24.83 [18.48, 34.65] | 23.96 [18.03, 32.26] | 23.56 [17.98, 32.84] | 23.48 [17.43, 32.45] | <0.001 |
| Albumin (g/L) | 44.89 [43.04, 46.75] | 44.70 [42.84, 46.56] | 44.59 [42.69, 46.53] | 44.61 [42.60, 46.41] | 0.001 |
| ALP (U/L) | 86.30 [71.70, 105.40] | 86.00 [72.00, 103.18] | 87.70 [73.30, 106.10] | 85.70 [70.40, 105.30] | 0.059 |
| AST (U/L) | 25.50 [21.00, 31.90] | 24.85 [20.50, 30.50] | 24.70 [20.50, 30.70] | 24.40 [20.50, 30.20] | <0.001 |
| CRP (median [IQR]) | 1.90 [0.94, 4.02] | 2.03 [0.97, 4.24] | 2.02 [0.96, 4.29] | 1.88 [0.88, 3.98] | 0.014 |
| Serum calcium (mmol/L) | 2.39 [2.32, 2.46] | 2.38 [2.32, 2.45] | 2.38 [2.32, 2.45] | 2.38 [2.32, 2.45] | 0.042 |
| Cholesterol (mmol/L) | 4.34 [3.76, 5.03] | 4.33 [3.73, 4.98] | 4.23 [3.65, 4.96] | 4.30 [3.72, 5.02] | 0.004 |
| Creatinine (umol/L) | 73.00 [62.00, 85.80] | 73.20 [62.12, 86.57] | 72.80 [61.32, 86.10] | 72.10 [60.40, 84.70] | 0.042 |
| γGGT (U/L) | 36.10 [24.20, 59.40] | 35.30 [23.83, 57.38] | 35.20 [23.80, 55.90] | 33.80 [23.30, 54.70] | 0.048 |
| HbA1c (%) | 51.30 [44.00, 60.80] | 51.70 [44.30, 61.00] | 51.60 [44.00, 60.50] | 51.60 [43.90, 61.50] | 0.733 |
| Phosphate (mmol/L) | 1.14 [1.03, 1.26] | 1.14 [1.03, 1.25] | 1.15 [1.03, 1.26] | 1.16 [1.05, 1.27] | <0.001 |
| Triglycerides (mmol/L) | 1.91 [1.30, 2.78] | 1.82 [1.24, 2.61] | 1.80 [1.28, 2.51] | 1.72 [1.16, 2.51] | <0.001 |
| Blood urate (umol/L) | 5.69 [4.76, 6.87] | 5.75 [4.78, 6.85] | 5.63 [4.67, 6.85] | 5.48 [4.54, 6.67] | <0.001 |
| Vitamin D (nmol/L) | 43.00 [29.30, 59.00] | 41.05 [28.20, 58.90] | 38.50 [26.50, 54.80] | 35.00 [23.70, 50.80] | <0.001 |
| Hypertension (%) |  |  |  |  | 0.016 |
| No | 738 (30.5) | 709 (29.3) | 643 (26.6) | 675 (27.9) |  |
| Yes | 1679 (69.5) | 1709 (70.7) | 1775 (73.4) | 1742 (72.1) |  |
| Chronic renal failure (%) |  |  |  |  | 0.413 |
| No | 2284 (94.5) | 2269 (93.8) | 2269 (93.8) | 2290 (94.7) |  |
| Yes | 133 (5.5) | 149 (6.2) | 149 (6.2) | 127 (5.3) |  |
| Course of diabetes (%) |  |  |  |  | 0.828 |
| <3 | 1149 (47.5) | 1151 (47.6) | 1121 (46.4) | 1097 (45.4) |  |
| 3~5 | 463 (19.2) | 455 (18.8) | 457 (18.9) | 484 (20.0) |  |
| 5~10 | 674 (27.9) | 679 (28.1) | 689 (28.5) | 693 (28.7) |  |
| ≥10 | 131 (5.4) | 133 (5.5) | 151 (6.2) | 143 (5.9) |  |
| Annual income (%) |  |  |  |  | <0.001 |
| £18,000~£30999 | 742 (30.7) | 864 (35.7) | 941 (38.9) | 889 (36.8) |  |
| £31000~£51999 | 601 (24.9) | 616 (25.5) | 556 (23.0) | 446 (18.5) |  |
| £52000~£100000 | 429 (17.7) | 346 (14.3) | 331 (13.7) | 309 (12.8) |  |
| ≥£100000 | 220 (9.1) | 170 (7.0) | 133 (5.5) | 172 (7.1) |  |
| unknow | 425 (17.6) | 422 (17.5) | 457 (18.9) | 601 (24.9) |  |

BMI: Body mass index; ALT: Alanine aminotransferase; AST: Aspartate aminotransferase; ALP: Alkaline phosphatase; γGGT: Gamma-glutamyl transferase; HbA1c: Glycated hemoglobin; CRP: C-reactive protein

Supplementary Table 3. Baseline characteristics of participants by PM₁₀ exposure quartiles.

| Variables | Q1 | Q2 | Q3 | Q4 | P-Value |
| --- | --- | --- | --- | --- | --- |
| Age (year) | 62.00 [57.00, 66.00] | 62.00 [56.00, 66.00] | 62.00 [56.00, 66.00] | 61.00 [55.00, 65.00] | <0.001 |
| Sex (%) |  |  |  |  | 0.01 |
| Female | 901 (37.5) | 920 (38.1) | 974 (40.4) | 1009 (41.6) |  |
| Male | 1500 (62.5) | 1495 (61.9) | 1437 (59.6) | 1414 (58.4) |  |
| Ethnic (%) |  |  |  |  | <0.001 |
| White | 2300 (95.8) | 2208 (91.4) | 2123 (88.1) | 1715 (70.8) |  |
| Mixed | 5 (0.2) | 12 (0.5) | 19 (0.8) | 23 (0.9) |  |
| Asian or Asian British | 64 (2.7) | 100 (4.1) | 156 (6.5) | 355 (14.7) |  |
| Black or Black British | 5 (0.2) | 46 (1.9) | 57 (2.4) | 181 (7.5) |  |
| Other | 27 (1.1) | 49 (2.0) | 56 (2.3) | 149 (6.1) |  |
| Alcohol (%) |  |  |  |  | <0.001 |
| Never | 169 (7.0) | 183 (7.6) | 236 (9.8) | 334 (13.8) |  |
| Previous | 191 (8.0) | 209 (8.7) | 228 (9.5) | 233 (9.6) |  |
| Current | 2029 (84.5) | 2003 (82.9) | 1923 (79.8) | 1821 (75.2) |  |
| unknow | 12 (0.5) | 20 (0.8) | 24 (1.0) | 35 (1.4) |  |
| Smoking (%) |  |  |  |  | <0.001 |
| No | 2147 (89.4) | 2129 (88.2) | 2110 (87.5) | 2043 (84.3) |  |
| Most or all days | 191 (8.0) | 199 (8.2) | 216 (9.0) | 265 (10.9) |  |
| Occasionally | 59 (2.5) | 71 (2.9) | 68 (2.8) | 84 (3.5) |  |
| unknow | 4 (0.2) | 16 (0.7) | 17 (0.7) | 31 (1.3) |  |
| BMI (Kg/m²) | 30.67 [27.32, 34.61] | 30.82 [27.49, 35.16] | 31.02 [27.45, 34.99] | 30.36 [27.01, 34.85] | 0.047 |
| ALT (U/L) | 24.08 [18.04, 33.37] | 24.46 [18.38, 33.09] | 24.23 [18.08, 33.29] | 23.21 [17.27, 32.03] | <0.001 |
| Albumin (g/L) | 44.80 [42.87, 46.67] | 44.81 [43.06, 46.75] | 44.66 [42.80, 46.43] | 44.52 [42.49, 46.38] | <0.001 |
| ALP (U/L) | 86.30 [71.90,104.80] | 87.40 [72.65, 106.25] | 86.10 [71.65, 104.70] | 85.90 [70.85, 104.50] | 0.251 |
| AST (U/L) | 25.00 [20.80, 30.80] | 25.20 [20.80, 31.20] | 24.90 [20.80, 31.05] | 24.40 [20.30, 30.10] | 0.017 |
| CRP (median [IQR]) | 1.87 [0.93, 4.00] | 2.03 [0.95, 4.16] | 1.94 [0.94, 4.17] | 1.99 [0.93, 4.22] | 0.478 |
| Serum calcium (mmol/L) | 2.38 [2.32, 2.45] | 2.39 [2.32, 2.45] | 2.38 [2.32, 2.45] | 2.38 [2.32, 2.44] | 0.112 |
| Cholesterol (mmol/L) | 4.29 [3.71, 4.96] | 4.33 [3.72, 5.04] | 4.27 [3.72, 4.92] | 4.32 [3.71, 5.05] | 0.211 |
| Creatinine (umol/L) | 73.40 [62.20, 86.80] | 73.20 [61.80, 86.30] | 73.00 [62.10, 86.20] | 71.80 [60.10, 84.00] | <0.001 |
| γGGT (U/L) | 35.50 [23.80, 56.20] | 35.70 [24.50, 57.10] | 35.20 [23.50, 57.40] | 34.00 [23.30, 55.70] | 0.208 |
| HbA1c (%) | 51.60 [44.40, 60.80] | 51.50 [43.70, 60.70] | 51.80 [44.00, 60.90] | 51.40 [43.90, 61.50] | 0.806 |
| Phosphate (mmol/L) | 1.13 [1.02, 1.25] | 1.15 [1.04, 1.26] | 1.14 [1.03, 1.25] | 1.16 [1.04, 1.27] | <0.001 |
| Triglycerides (mmol/L) | 1.84 [1.28, 2.66] | 1.84 [1.27, 2.66] | 1.83 [1.25, 2.59] | 1.71 [1.18, 2.48] | <0.001 |
| Blood urate (umol/L) | 5.72 [4.78, 6.93] | 5.68 [4.69, 6.83] | 5.63 [4.69, 6.79] | 5.53 [4.56, 6.73] | <0.001 |
| Vitamin D (nmol/L) | 40.90 [27.20, 57.40] | 40.80 [27.70, 57.70] | 39.20 [27.00, 55.85] | 37.00 [25.30, 53.50] | <0.001 |
| Hypertension (%) |  |  |  |  | 0.022 |
| No | 737 (30.7) | 705 (29.2) | 654 (27.1) | 666 (27.5) |  |
| Yes | 1664 (69.3) | 1710 (70.8) | 1757 (72.9) | 1757 (72.5) |  |
| Chronic renal failure (%) |  |  |  |  | 0.017 |
| No | 2260 (94.1) | 2247 (93.0) | 2287 (94.9) | 2300 (94.9) |  |
| Yes | 141 (5.9) | 168 (7.0) | 124 (5.1) | 123 (5.1) |  |
| Course of diabetes (%) |  |  |  |  | 0.173 |
| <3 | 1147 (47.8) | 1150 (47.6) | 1123 (46.6) | 1092 (45.1) |  |
| 3~5 | 456 (19.0) | 432 (17.9) | 469 (19.5) | 496 (20.5) |  |
| 5~10 | 672 (28.0) | 675 (28.0) | 694 (28.8) | 686 (28.3) |  |
| ≥10 | 126 (5.2) | 158 (6.5) | 125 (5.2) | 149 (6.1) |  |
| Annual income (%) |  |  |  |  | <0.001 |
| £18,000~£30999 | 805 (33.5) | 866 (35.9) | 864 (35.8) | 895 (36.9) |  |
| £31000~£51999 | 599 (24.9) | 573 (23.7) | 535 (22.2) | 508 (21.0) |  |
| £52000~£100000 | 388 (16.2) | 369 (15.3) | 341 (14.1) | 313 (12.9) |  |
| ≥£100000 | 195 (8.1) | 168 (7.0) | 168 (7.0) | 163 (6.7) |  |
| unknow | 414 (17.2) | 439 (18.2) | 503 (20.9) | 544 (22.5) |  |

BMI: Body mass index; ALT: Alanine aminotransferase; AST: Aspartate aminotransferase; ALP: Alkaline phosphatase; γGGT: Gamma-glutamyl transferase; HbA1c: Glycated hemoglobin; CRP: C-reactive protein

Supplementary Table 4. Associations of NO₂ and PM₁₀ quartiles with subtype-specific risks of diabetic microvascular complications: nephropathy, retinopathy, and neuropathy.

| Pollutant | Nephropathy | Retinopathy | Neuropathy |
| --- | --- | --- | --- |
| Subgroup | HR (95%CI) P-value | HR (95%CI) P-value | HR (95%CI) P-value |
| NO₂ |  |  |  |
| Q2 | 1.00 (0.85–1.18) P = 0.985 | 0.97 (0.82–1.15) P = 0.749 | 1.04 (0.88–1.23) P = 0.638 |
| Q3 | 1.15 (0.98–1.35) P = 0.086 | 1.06 (0.90–1.25) P = 0.481 | 1.08 (0.92–1.27) P = 0.352 |
| Q4 | 1.30 (1.11–1.52) P = 0.001 | 1.21 (1.03–1.42) P = 0.021 | 1.15 (0.98–1.35) P = 0.089 |
| PM₁₀ |  |  |  |
| Q2 | 1.24 (1.05–1.46) P = 0.012 | 1.18 (1.00–1.39) P = 0.051 | 1.16 (0.98–1.37) P = 0.082 |
| Q3 | 1.35 (1.15–1.58) P<0.001 | 1.26 (1.07–1.48) P = 0.006 | 1.22 (1.04–1.43) P = 0.015 |
| Q4 | 1.50 (1.28–1.76) P<0.001 | 1.40 (1.19–1.65) P<0.001 | 1.31 (1.11–1.54) P = 0.002 |
